# Cisplatin resistance in oral squamous cell carcinoma: mechanisms, reversal strategies, and emerging technologies

**DOI:** 10.3389/fphys.2026.1790855

**Published:** 2026-04-15

**Authors:** Yuanxin Shi, Wenjin Liao, Guohui Bai, Bin Chen

**Affiliations:** 1 Key Laboratory of Oral Disease Research of the Education Department of Guizhou Province, School of Stomatology, Zunyi Medical University, Zunyi, China; 2 Zunyi Laboratory of Oral Disease Research, Zunyi Medical University, Zunyi, China; 3 The First Clinical College, Zunyi Medical University, Zunyi, China

**Keywords:** chemotherapy resistance, cisplatin, emerging technologies, oral squamous cell carcinoma, tumor microenvironment, targeted therapy

## Abstract

Cisplatin remains a first-line chemotherapeutic agent in the treatment of oral squamous cell carcinoma (OSCC). However, the efficacy of cisplatin is frequently compromised by the development of drug resistance. This review systematically examines the multidimensional mechanisms underlying cisplatin resistance in OSCC and the corresponding strategies to overcome this resistance. Mechanisms of chemoresistance involve complex, multi-layered molecular networks, encompassing dysregulation of key gene expression and signaling pathways, epigenetic remodeling, metabolic reprogramming, evasion of regulated cell death, acquisition of epithelial–mesenchymal transition (EMT) and cancer stem cell (CSC) properties, as well as the formation of an immunosuppressive tumor microenvironment (TME). In response to these challenges, multimodal combinatorial approaches are being developed, including small-molecule inhibitors targeting specific resistance nodes, nanotechnology-based targeted drug delivery systems, combination therapies with immune checkpoint inhibitors, and interventions targeting metabolic vulnerabilities. Furthermore, emerging technologies are enabling more precise strategies: patient-derived organoids provide a platform for individualized drug sensitivity testing; single-cell sequencing allows for dissection of cellular heterogeneity within resistant populations and the interactions of these populations with the microenvironment; and artificial intelligence (AI) aids in predictive model building and drug discovery by integrating multi-omics data. In summary, a comprehensive understanding of the systems biology of cisplatin resistance, integrated with novel research paradigms such as nanotechnology, immunotherapy, metabolic targeting, organoid models, single-cell technologies, and AI, will be pivotal for developing personalized combination therapies to ultimately overcome cisplatin resistance in OSCC.

## 1 Introduction

Cisplatin, a cornerstone chemotherapeutic agent in the treatment of OSCC, exerts its antitumor effects by forming DNA cross-links (Wang and Lippard, 2005). Such DNA lesions induce DNA crosslinking and strand breaks, leading to cell cycle arrest and apoptosis (Wang et al., 2012). Despite the established clinical utility of cisplatin, the clinical efficacy of cisplatin is frequently compromised by the development of drug resistance, a major challenge given the aggressive nature and high recurrence rate of OSCC (Rabik and Dolan, 2007; Galluzzi et al., 2014; Aminuddin et al., 2024; Oh et al., 2024). Drug resistance not only contributes to treatment failure but is also associated with significantly poorer patient survival (Luo et al., 2015; Wang et al., 2025a). Therefore, elucidating the mechanisms underlying both the therapeutic action and the resistance to cisplatin is of critical clinical importance.

The current standard of care for locally advanced OSCC often involves multimodal strategies integrating surgery, radiotherapy, and chemotherapy (Bera et al., 2023). Among these, platinum-based combination regimens, particularly the TPF protocol (docetaxel, cisplatin, and 5-fluorouracil), are widely employed in induction chemotherapy or adjuvant settings to improve survival outcomes and organ preservation (Vermorken et al., 2007). Although these platinum-based regimens have demonstrated clinical benefits, their efficacy is frequently constrained by cumulative toxicities (e.g., myelosuppression, nephrotoxicity, severe oral mucositis) (Oun et al., 2018) and the inevitable emergence of intrinsic or acquired drug resistance (Zhong et al., 2015; Fayette et al., 2016). Such limitations underscore the critical and unmet need for deeper mechanistic insights and the development of novel, targeted therapeutic strategies.

Consistent with this notion, rapid advances in molecular biology techniques have greatly deepened the understanding of cisplatin resistance mechanisms, revealing an increasingly complex picture involving multi-level molecular regulatory networks and cellular processes (Rabik and Dolan, 2007; Galluzzi et al., 2014; Aminuddin et al., 2024). In OSCC, dysregulated expression of several key proteins, such as Naa10p (Sun et al., 2021) and *MTMR6* (Lee et al., 2025), has been shown to be closely associated with cisplatin resistance in cell-based assays and correlative clinical studies. At the signaling pathway level, aberrant activation of critical pathways including the Hippo pathway has also been implicated in mediating resistant phenotypes (Zeng and Dong, 2021). Additionally, non-coding RNAs, such as long non-coding RNAs (e.g., *GAS5*) (Yuan et al., 2022) and circular RNAs (e.g., *circ-ILF2*) (Wu et al., 2023), can modulate cisplatin sensitivity through intricate regulatory networks and may further influence immune cell functions within the TME. Collectively, these observations elucidate the mechanisms underlying cisplatin resistance from multiple perspectives, laying a theoretical foundation for developing strategies to reverse resistance.

In addition to transcriptional and signaling alterations, metabolic reprogramming plays a pivotal role in the emergence and progression of cisplatin resistance (Chen et al., 2022). Experimental evidence from metabolomic profiling of OSCC cell lines indicates that even short-term cisplatin exposure can trigger dynamic metabolic responses in OSCC cells, leading to specific changes in various metabolic pathways and metabolites, which may form the basis of early adaptive reactions in OSCC cells (Chen et al., 2022). In cisplatin-resistant OSCC cell lines established through chronic drug exposure, metabolic remodeling becomes more pronounced; to meet the increased energy demands of rapid proliferation, resistant OSCC cells often enhance alternative energy-producing pathways, such as glycolysis (Aminuddin et al., 2020). Beyond energy metabolism, abnormal activation of specific metabolic pathways has been shown to directly drive resistance in OSCC (Torres et al., 2024). Moreover, evasion of ferroptosis confers a survival advantage on OSCC cells and helps maintain the resistant state by regulating lipid metabolism and intracellular iron homeostasis (Wu et al., 2024).

In response to the challenge of cisplatin resistance, nanotechnology-based targeted delivery strategies and multimodal combination therapies have emerged as important research directions (Liu et al., 2025). Nanocarrier systems, such as hyaluronic acid nanogels (Gao et al., 2024) and metal-organic frameworks (An et al., 2024), can effectively enhance tumor targeting and accumulation of cisplatin while reducing systemic toxicity. Meta-analyses have further supported the potential of these platforms to improve efficacy and minimize adverse effects (Ranasinghe et al., 2024). Beyond delivery systems, integrating cisplatin with photothermal therapy (PPT) (Liu et al., 2025), immune checkpoint inhibitors (Jia et al., 2024), or specific pathway inhibitors (e.g., those targeting ferroptosis or signal transduction) (Wang et al., 2025; Zhang et al., 2025) has shown promising synergistic effects in reversing resistance. Moreover, multifunctional nano-platforms that combine chemotherapy, immunomodulation, and physiodynamic therapy offer innovative solutions for overcoming drug resistance (An et al., 2024).

While traditional mechanisms of cisplatin resistance continue to be explored, emerging technologies are transforming research paradigms in the understanding of cisplatin resistance. For instance, patient-derived organoid models closely replicate tumor heterogeneity and the microenvironment, providing a crucial platform for individualized drug sensitivity testing and combination screening *in vitro* (Takagi et al., 2024; Nitschke et al., 2025). Single-cell sequencing offers unprecedented resolution to uncover the evolution of tumor cell heterogeneity, the emergence of rare resistant subpopulations, and the reprogramming of immune and stromal cells in the TME, thereby enabling precise mapping of resistant cell states (Liu et al., 2024; Huang et al., 2025). Furthermore, artificial intelligence and machine learning can integrate multi-omics and clinical data to construct high-accuracy predictive models for early risk identification, prognosis assessment, and intelligent discovery of novel biomarkers and resistance-reversing agents (Poirion et al., 2021; Fountzilas et al., 2025). The coordinated application of these technologies is advancing the study of cisplatin resistance in OSCC from population-level descriptions toward a new era of precision intervention based on cellular atlases and individualized models.

In summary, although cisplatin remains a highly effective agent in OSCC treatment, the clinical utility of this platinum compound is often limited by multifaceted resistance mechanisms. Systematic elucidation of these mechanisms provides a theoretical foundation for personalized and precision therapy in clinical practice. Notably, the integration of combination therapies, nanomaterial-based delivery, and emerging research paradigms—such as organoids, single-cell sequencing, and artificial intelligence—opens new pathways and brings renewed hope for overcoming resistance and enhancing therapeutic outcomes.

## 2 The anti-tumor mechanism of cisplatin and its application in OSCC

### 2.1 Overview of the mechanism of action of cisplatin

As a first-line chemotherapeutic agent for OSCC, cisplatin exerts antitumor effects through a multi-step process, starting with cellular entry and culminates in the induction of regulated cell death (Rabik and Dolan, 2007; Galluzzi et al., 2014). Cisplatin enters cells through both passive diffusion and active transport mediated by copper transporters, particularly copper transporter 1 (*CTR1*) (Holzer et al., 2006). Once inside the cell, the relatively high chloride concentration in the extracellular milieu maintains cisplatin in a neutral state. However, upon entering the cytoplasm where chloride concentration is low, cisplatin undergoes aquation—a process in which chloride ligands are replaced by water molecules, and the compound becomes a highly reactive, positively charged electrophile (Thompson and Joy, 2024). This activated form readily reacts with nucleophilic sites on DNA, preferentially binding to the N7 position of guanine bases to form intrastrand and interstrand crosslinks, which distort the DNA helix (Jamieson and Lippard, 1999). These DNA lesions trigger a multifaceted cellular response. The formation of cisplatin-DNA adducts not only directly interferes with DNA replication and transcription but can also induce oxidative stress through the generation of reactive oxygen species (ROS), further amplifying cellular damage (Marullo et al., 2013). Subsequently, the resulting DNA damage is recognized by sensor proteins that activate the DNA damage response (DDR) signaling cascade. This cascade involves phosphorylation of checkpoint kinases, specifically ataxia telangiectasia mutated (*ATM*) and *ATM* and Rad3-related (*ATR*), which subsequently activate downstream effectors including *CHK1*, *CHK2*, and *p53* (Lindemann et al., 2018). Activation of p53 orchestrates a transcriptional program that can lead to cell cycle arrest—allowing time for DNA repair—or, if damage is irreparable, trigger regulated cell death (Jamieson and Lippard, 1999). Ultimately, the cytotoxicity triggered by this process in OSCC is mainly manifested through the induction of apoptosis.

In addition, recent studies indicate that cisplatin can activate caspase cascades via multiple pathways to execute the apoptotic program in OSCC. These include the intrinsic (mitochondrial) pathway, involving key regulators such as the Bcl-2 family proteins. Specifically, cisplatin-based regimens have been shown to downregulate anti-apoptotic *Bcl-xL* (Alam and Mishra, 2021) and *Bcl-2* while upregulating pro-apoptotic *Bax* and *p53* (Al-Serwi et al., 2020; Cabral et al., 2025). Furthermore, the extrinsic (death receptor) pathway may also contribute, as evidenced by the activation of caspase-8 observed in OSCC cells treated with cisplatin in combination with other agents (Cabral et al., 2025). This multi-pathway engagement ultimately leads to the cleavage of cellular substrates and apoptotic cell death (Al-Serwi et al., 2020; Cabral et al., 2025). Furthermore, recent research has revealed that cisplatin can also induce ferroptosis. Key regulatory molecules such as *CAV1*, *TPI1*, and *AEBP1* play essential roles in this process and are closely associated with drug resistance (Zhou et al., 2022; Wang et al., 2025; Zhang et al., 2025).

**Figure 1:**
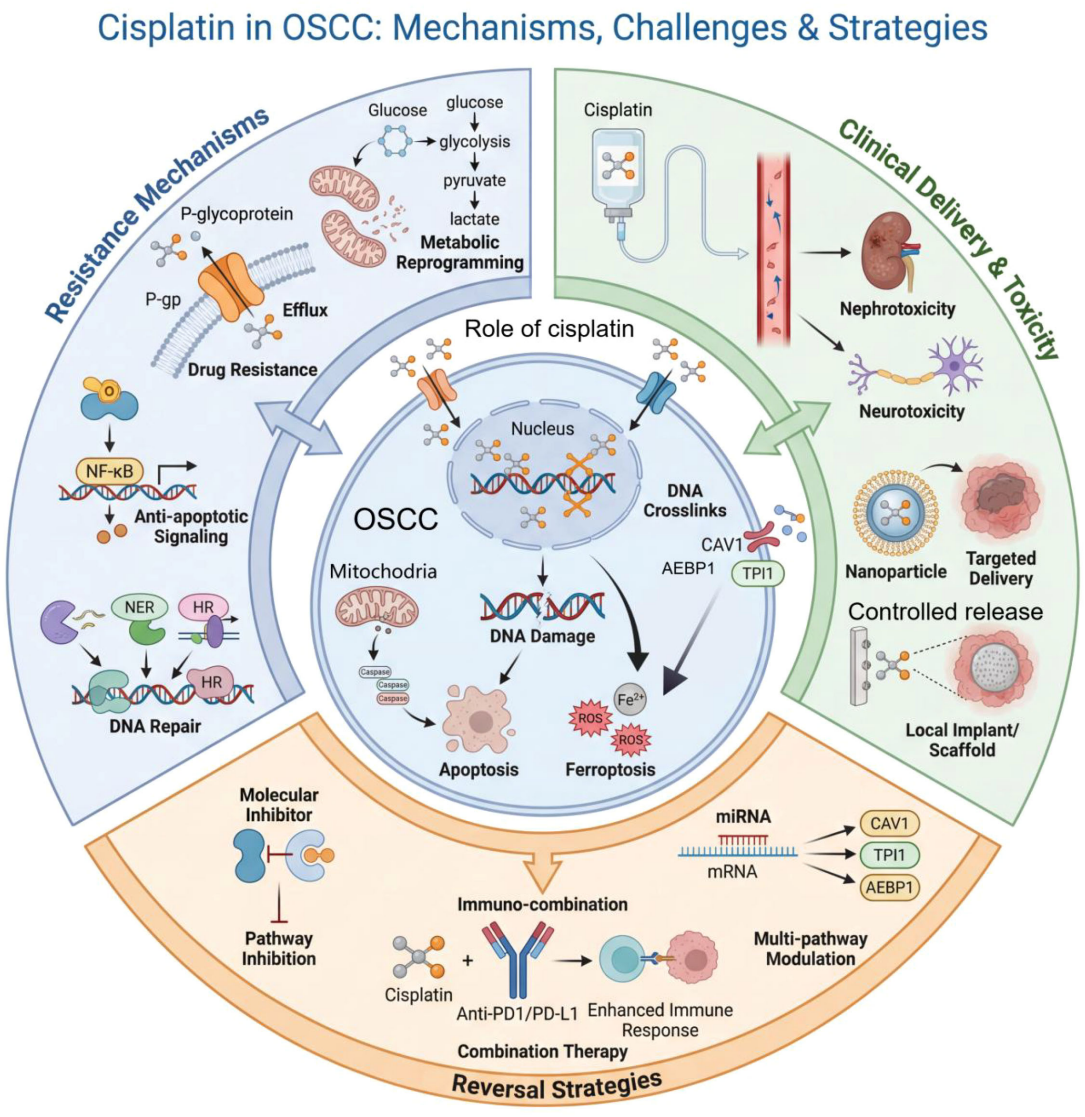
The multifaceted role of cisplatin in OSCC. This schematic integrates the drug's anti-tumor mechanisms with the challenges of resistance and clinical toxicity, and highlights potential strategies to enhance its efficacy. The core mechanism involves cisplatin-induced DNA damage, triggering apoptosis and ferroptosis—a process modulated by CAV1, TPI1, and AEBP1. Primary resistance mechanisms include drug efflux via P-glycoprotein (P-gp), metabolic reprogramming, NF-κB-mediated survival signaling, and enhanced DNA repair through NER and HR. Clinically, systemic delivery is limited by nephro- and neurotoxicity, prompting the development of targeted nanoparticles and local delivery systems. Reversal strategies to combat resistance encompass molecular inhibitors, combination with immune checkpoint inhibitors (anti-PD-1/PD-L1), and miRNA-based therapies that target key regulators like CAV1, TPI1, and AEBP1.

In summary, cisplatin primarily triggers multiple forms of cell death, including apoptosis and ferroptosis, by inducing DNA damage. Cisplatin’s therapeutic efficacy is finely modulated by drug transport, metabolic adaptation, and intracellular signaling pathways. Combined interventions targeting these regulatory mechanisms represent a key direction for improving chemotherapy outcomes and overcoming resistance in cancer cells. (**Figure 1****).**

### 2.2 Current status of clinical application of cisplatin therapy

Clinically, cisplatin, a platinum-based chemotherapeutic agent, serves as a cornerstone in the treatment of OSCC. The antitumor activity of this platinum agent is mediated primarily through the formation of DNA adducts, which distort the DNA helix, inhibit replication and transcription, and ultimately trigger cell cycle arrest and apoptosis in OSCC (Jamieson and Lippard, 1999). In clinical practice, cisplatin is widely used in chemoradiotherapy for advanced or recurrent OSCC, with standard dosing regimens including 100 mg/m² every three weeks or 40 mg/m² weekly (Kiyota et al., 2022), it also constitutes a key component of various induction or adjuvant chemotherapy regimens, often in combination with agents such as paclitaxel and 5-fluorouracil (Sahoo et al., 2024). For instance, the TPF regimen has been shown to significantly improve overall survival and progression-free survival in locally advanced head and neck squamous cell carcinoma (HNSCC) compared to cisplatin plus 5-fluorouracil alone (Lorch et al., 2011), with a median overall survival of 70.6 months versus 34.8 months (HR = 0.74, 95% CI: 0.58–0.94) and a 5-year survival rate of 52% versus 42%. Alternatively, nanotechnology-based targeted delivery systems (e.g., magnetic nanoplatforms) can achieve synergistic effects by combining chemotherapy with PPT, thereby improving antitumor outcomes in both *in vitro* and *in vivo* models of OSCC (Liu et al., 2025). Local delivery systems (e.g., PRV111), on the other hand, can significantly increase intratumoral drug concentrations while avoiding severe systemic toxicity, as demonstrated in animal models and subsequently validated in a phase 1/2 clinical trial for OCSCC patients (Goldberg et al., 2022).

Despite these advances, the clinical use of cisplatin has long been constrained by two major challenges: intrinsic or acquired drug resistance and dose-limiting toxicities. Resistance to cisplatin in OSCC involves complex, multi-layered mechanisms, including altered drug metabolism (e.g., dysregulation of transport proteins) (Sun et al., 2021), enhanced DNA repair, evasion of cell death (e.g., resistance to apoptosis and ferroptosis) (Wang et al., 2025; Zhang et al., 2025), epigenetic and gene expression regulation (e.g., downregulation of *Naa10p* and *MTMR6*) (Sun et al., 2021; Lee et al., 2025), metabolic reprogramming (e.g., mitochondrial DNA alterations and enhanced glycolysis) (Aminuddin et al., 2020; Chen et al., 2022), as well as adaptive changes in the TME (e.g., activation of autophagy and chromatin remodeling) (Oh et al., 2024). These mechanisms collectively contribute to diminished therapeutic response. Historically, randomized phase III trials have reported objective response rates for cisplatin monotherapy in recurrent/metastatic HNSCC (R/M HNSCC) ranging from 10% to 17% (Jacobs et al., 1992; Catimel et al., 1994), underscoring the need for more effective combination approaches. Furthermore, cisplatin-induced toxicities—such as nephrotoxicity, neurotoxicity, gastrointestinal reactions, and myelosuppression—significantly compromise patient tolerance and quality of life, limiting long-term administration at optimal doses (Matsumoto et al., 2021; Sahoo et al., 2024).

In response to these challenges, researchers are actively developing multifaceted reversal strategies (de Bakker et al., 2024). At the molecular level, targeting specific signaling pathways has proven effective in restoring sensitivity in resistant cells (Xie et al., 2021; Kawabe et al., 2025). Simultaneous modulation of multiple cytoprotective pathways using specific microRNAs also offers a promising approach to sensitize OSCC to cisplatin. For example, *miR-634* targets multiple cytoprotective genes, including *cIAP1*, thereby enhancing cisplatin efficacy in both *in vitro* and *in vivo* models of OSCC (Tran et al., 2022). Regarding treatment modalities, neoadjuvant immunochemotherapy regimens combining cisplatin with specific immune checkpoint inhibitors have demonstrated high pathological response rates and potential survival benefits in locally advanced OSCC, representing a major focus of current clinical research. These include toripalimab (Huang et al., 2023), camrelizumab (Xiang et al., 2025), and tislelizumab (Wu et al., 2023), all of which are PD-1 inhibitors that have shown promising results when combined with cisplatin-based chemotherapy in recent trials. For instance, the phase III KEYNOTE-048 trial demonstrated that the addition of pembrolizumab to cisplatin-based chemotherapy significantly improved overall survival compared with cetuximab plus chemotherapy in patients with R/M HNSCC, including oral cavity cancer, with a hazard ratio of 0.71 (95% CI, 0.59 to 0.85) in the total population and 0.64 (95% CI, 0.53 to 0.78) in the PD-L1 combined positive score (CPS) ≥1 population. The objective response rate was 36.3% in both arms, with more durable responses observed in the pembrolizumab-containing arm (Harrington et al., 2023). Furthermore, innovative formulations such as scaffold-based local sustained-release systems are designed to achieve more precise tumor-targeted drug delivery, aiming to enhance efficacy while minimizing systemic exposure and toxicity (Saberian et al., 2024).

In summary, cisplatin remains integral to the comprehensive management of OSCC, yet the effectiveness of this agent is substantially limited by resistance and toxicity. Future efforts to overcome these barriers will likely rely on a deeper understanding of resistance networks, the development of novel combination therapies—particularly immunocombination strategies—and advances in drug delivery technologies, collectively paving the way toward more effective and safer therapeutic options for patients with OSCC. (**Figure 1**).

## 3 Mechanisms of cisplatin resistance

The development of cisplatin resistance in OSCC is increasingly recognized as a dynamic, multi-stage process rather than a binary outcome. Emerging evidence highlights drug-tolerant persister cells (DTPs) as a critical origin of acquired resistance (Ling et al., 2026). DTPs are defined as a subpopulation of cancer cells that survive initial cytotoxic stress by entering a reversible, slow-cycling state, accompanied by distinct epigenetic and metabolic reprogramming (Wu et al., 2024; Li et al., 2026). These persister cells serve as a cellular reservoir from which genetically stable, fully resistant clones eventually emerge under sustained therapeutic pressure (Rabik and Dolan, 2007). Several reports have demonstrated that DTPs contribute significantly to chemotherapy failure and tumor relapse in various cancers, including OSCC (Álvarez-Varela et al., 2022; Li et al., 2025; Ling et al., 2026). Targeting DTPs before they evolve into fully resistant clones is therefore essential for eradicating resistance at its inception.

These diverse adaptive mechanisms operate across multiple biological levels and can be broadly categorized into several interconnected classes: (1) reduced drug accumulation; (2) enhanced drug detoxification; (3) augmented DNA repair capacity (e.g., via nucleotide excision repair); (4) evasion of apoptosis; (5) epigenetic and transcriptional reprogramming; and (6) activation of alternative survival pathways. In the following sections, key molecules involved in each category are discussed in detail.

### 3.1 Multi-level molecular mechanisms of cisplatin resistance

Clinically, the efficacy of cisplatin is intricately regulated by intracellular pharmacokinetics and the cellular state. At the cellular level, resistance mechanisms manifest through several interconnected processes that directly modulate the drug’s therapeutic efficacy. Transport-mediated resistance, such as altered expression of membrane transporters—particularly the efflux pump *P-gp*—can reduce intracellular cisplatin accumulation, representing one of the fundamental mechanisms underlying resistance (Sun et al., 2021). Additionally, metabolic reprogramming in OSCC cells significantly affects drug sensitivity. Resistant cells often exhibit mitochondrial DNA alterations and a shift in energy metabolism toward glycolysis, thereby diminishing cisplatin-induced cytotoxicity (Aminuddin et al., 2020; Chen et al., 2022). At the signaling pathway level, activation of pathways such as *NF-κB* exerts anti-apoptotic effects, promoting OSCC cell survival and resistance, whereas inhibition of these pathways can enhance cisplatin-triggered cell death (Chen et al., 2020).

These diverse adaptive responses are driven by specific molecular alterations. The following sections detail the key molecules and pathways involved in reduced drug accumulation, evasion of cell death, and epigenetic reprogramming.

Reduced drug accumulation and enhanced detoxification: dysregulation of drug transporters and metabolic enzymes contributes to decreased intracellular cisplatin levels. Efflux pump overexpression, such as *P−gp*, reduces drug retention; for instance, in cisplatin-resistant OSCC cell lines, downregulation of *Naa10p* leads to increased *P−gp* levels, and restoring *Naa10p* expression resensitizes cells to cisplatin (Sun et al., 2021). Conversely, reduced drug influx also plays a critical role. The primary cisplatin uptake *CTR1* has been shown to be downregulated in resistant cells, limiting intracellular drug availability and further promoting resistance (Ogawa et al., 2024). Additionally, *MTMR6* expression positively correlates with cisplatin sensitivity; this phosphatase is negatively regulated by *miR−544a*, and reduced *MTMR6* expression promotes resistance, potentially through modulation of phosphatidylinositol metabolism (Lee et al., 2025).

Deregulation of cell death pathways and survival signaling: RRBP1, identified through integrated omics profiling, is upregulated in cisplatin-resistant cells and tumors from non-responder patients; its knockout or pharmacological inhibition restores cisplatin-mediated cell death and significantly reduces tumor burden in xenograft models (Shriwas et al., 2021). *IGF2BP1* is markedly upregulated in resistant cells; its knockdown reduces resistance, while overexpression dramatically increases cisplatin resistance through activation of the *Akt* signaling pathway (Xie et al., 2021). The histone methyltransferase *EZH2* accumulates in cisplatin-resistant and CSC populations; it epigenetically silences *APC* by binding its promoter, thereby activating Wnt/β-catenin signaling, promoting CSC accumulation and chemoresistance; combined inhibition of *EZH2* and β-catenin effectively reduces tumor volume and CSC population *in vivo* (Milan et al., 2023). Additionally, upregulation of the cell−cycle kinase *MASTL* inhibits the PP2A/B55 phosphatase via phosphorylation of *ENSA*, thereby disrupting the DNA damage response checkpoint, and treatment with MASTL inhibitor GKI−1 effectively reverses resistance (Gouttia et al., 2022).

Epigenetic and transcriptional reprogramming: These genetic and signaling alterations are embedded within a systematic network of transcriptomic and epigenetic changes. Transcriptomic analyses reveal that resistant cells exhibit numerous differentially expressed genes enriched in pathways such as TNF and MAPK signaling, with *TNF*, *TGFB1*, and *EGF* identified as core resistance−related genes (Wang et al., 2025). Such global reprogramming is tightly regulated by multi−layered epigenetic mechanisms. Histone deacetylase *HDAC6* accumulates in resistant cells and CSCs, maintaining the resistant phenotype by modulating oxidative stress and the DNA damage response (Tavares et al., 2022); *HDAC6* accumulation is accompanied by downregulation of *HDAC1/2* and upregulation of EMT-related genes such as *ZEB1* and *BMI−1* (Lima De Oliveira et al., 2023). DNA methylation leads to silencing of the tumor suppressor *HOXA5*, and restoring *HOXA5* expression significantly sensitizes cells to cisplatin (Chen et al., 2025). The m^6^ A methyltransferase *METTL3* drives resistance by forming a positive feedback loop or stabilizing oncogene mRNAs such as *c−Myc* (Wang et al., 2022; Liu et al., 2025). Integrated omics studies have also identified novel resistance−associated factors such as *S100A2* (Inukai et al., 2020).

**Figure 2:**
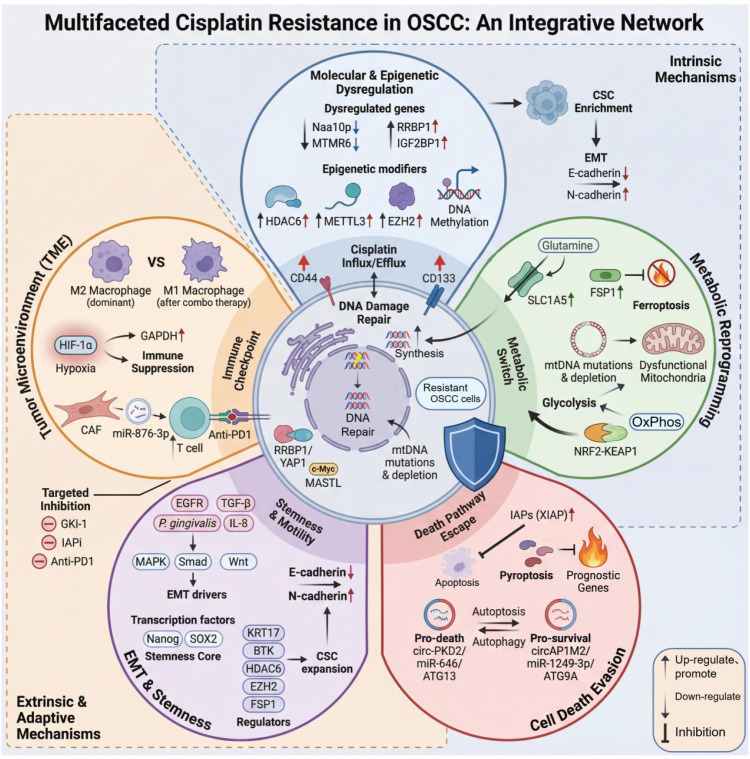
An integrative network of multifaceted cisplatin resistance in OSCC. This schematic illustrates the complex, interconnected mechanisms driving cisplatin resistance in OSCC, organized around a resistant cell core influenced by intrinsic dysregulation, extrinsic adaptive processes, and theTME. The central resistant OSCC cell is defined by enhanced DNA repair, altered drug influx/efflux (via CD44 and CD133), and mitochondrial DNA (mtDNA) mutations. Intrinsic drivers of resistance include molecular and epigenetic dysregulation—such as altered expression of Naa10p, MTMR6, RRBP1, IGF2BP1, and epigenetic modifiers (HDAC6, METTL3, EZH2, DNA methylation)—that promote CSC enrichment and EMT. This is coupled with metabolic reprogramming, featuring glutamine addiction (SLC1A5), FSP1-mediated ferroptosis evasion, a switch from oxidative phosphorylation to glycolysis, and NRF2-KEAP1 pathway activation. Extrinsic and adaptive mechanisms involve evasion of cell death via IAP upregulation (XIAP) and context-dependent autophagy regulation (e.g., circ-PKD2/miR-646/ATG13 axis), alongside EMT and stemness driven by external signals (EGFR, TGF-β, IL-8, P. gingivalis) that activate key pathways (MAPK, Smad, Wnt) and core transcription factors (Nanog, SOX2). The TME further fuels resistance through hypoxia (HIF-1α), M2 macrophage polarization, CAF-derived exosomal miR-876-3p, and immune checkpoint molecules (PD-1/PD-L1). Together, these layers form a dynamic network originating from DTPs and evolving into stable resistance, with potential intervention points including GKI-1 (MASTL inhibitor), IAP inhibitors, and anti-PD-1 immunotherapy.

In summary, cisplatin resistance constitutes a complex adaptive network driven by specific genes, precisely regulated through epigenetic mechanisms, and ultimately executed via reduced drug accumulation, evasion of apoptosis, activation of core survival pathways (e.g., Wnt/β−catenin, *Akt*), and disruption of cell−cycle checkpoints. Targeting key nodes within this network—such as *RRBP1*/YAP1, *HDAC6*, *METTL3*, *EZH2*/β−catenin, and *MASTL*—represents promising therapeutic strategies for reversing cisplatin resistance. (**Figure 2**).

### 3.2 Metabolic reprogramming and mitochondrial function

Cisplatin resistance in OSCC is closely associated with profound remodeling of cellular metabolic states (Chen et al., 2022). Through systematic metabolic reprogramming and mitochondrial dysfunction, cancer cells establish key adaptive mechanisms underlying drug resistance.

This process is first reflected in the increased reliance on specific metabolic pathways. Upregulation of the neutral amino acid transporter *SLC1A5* enhances glutamine uptake, supporting tumor biosynthesis and counteracting oxidative stress, thereby promoting resistance; inhibition of *SLC1A5* can restore cisplatin sensitivity by inducing multiple forms of cell death, including apoptosis, autophagy, and ferroptosis (Liu et al., 2024). Concurrently, reprogramming of lipid metabolism is directly linked to ferroptosis resistance. The ferroptosis suppressor protein FSP1 is highly expressed in resistant and recurrent OSCC tissues, where this protein protects tumor cells—particularly drug−tolerant stem−like subpopulations—from ferroptosis by regulating iron homeostasis and lipid metabolism, thereby driving resistance, genetic or pharmacological targeting of *FSP1* in cell line and xenograft models can reverse this phenotype (Wu et al., 2024).

Mitochondrial dysfunction represents another core aspect of metabolic reprogramming. Compared to their parental counterparts, cisplatin-resistant OSCC cell lines frequently exhibit reduced mitochondrial DNA (mtDNA) content, along with specific point mutations and epigenetic alterations (Aminuddin et al., 2024). Such abnormalities may stem from metabolic switching—from oxidative phosphorylation toward glycolysis—as cells adapt to chemotherapy−induced stress. This metabolic shift is accompanied by suppressed mitochondrial respiration, ultimately leading to mtDNA depletion and impairing cisplatin’s ability to induce cytotoxicity via oxidative stress (Aminuddin et al., 2020). The patterns of mtDNA alterations vary across different cell lines, indicating tumor−heterogeneity in the underlying mechanisms. In SAS-derived resistant cells (SAS-R), prolonged cisplatin exposure induced enrichment of the heteroplasmic m.3910G > C mutation in *MT−ND1*, reduced mtDNA content, and hypermethylation of CpG sites within the D−loop, *MT−ND4*, and *MT−ND5*. In contrast, H103-derived resistant cells (H103-R) showed no mutation enrichment or mtDNA content change, but displayed distinct hypermethylation in *MT−RNR2*, *MT−CO1*, and *MT−CYB* (Aminuddin et al., 2024).

Furthermore, key stress−response signaling axes are deeply involved in mediating cisplatin resistance and promoting distant metastasis (Osman et al., 2023). The *NRF2−KEAP1* antioxidant pathway, for instance, is often aberrantly activated in resistant cells through acquired *KEAP1* mutations or epigenetic reprogramming. Upregulation of *NRF2* downstream target genes—including those governing glutamine metabolism (e.g., *GLS1*)—enhances cellular antioxidant defenses and metabolic plasticity, thereby not only contributing to chemotherapy resistance but also correlating with an increased risk of distant metastasis in preclinical models and patient cohorts (Osman et al., 2023).

Importantly, these metabolic adaptations are particularly pronounced in DTPs—a reversible slow-cycling state that serves as a precursor to acquired resistance (Nie and Hu, 2024). DTPs survive initial cisplatin exposure by entering quiescence, a process tightly coupled with metabolic plasticity, including enhanced mitochondrial dynamics and the flexible utilization of oxidative phosphorylation or glycolysis in response to microenvironmental cues (Li et al., 2023). This metabolic remodeling allows DTPs to evade cisplatin-induced cytotoxicity, thereby creating a temporal window for the accumulation of genetic alterations that ultimately drive stable resistance. Notably, the protective function of *FSP1* in lipid metabolism is also evident in DTPs, where this protein safeguards cancer stem-like subpopulations from ferroptosis (Wu et al., 2024). Therefore, targeting the unique metabolic dependencies of DTPs—such as the distinct mitochondrial states of DTPs or *FSP1*-mediated lipid metabolism—represents a promising strategy to intercept resistance at its earliest stage.

In summary, cisplatin resistance in OSCC arises from a multi-layered metabolic adaptation process, characterized by reprogrammed substrate utilization, evasion of specific cell death pathways, mitochondrial dysfunction, and activation of key stress-responsive signaling cascades. Collectively, these interconnected alterations establish a metabolic landscape that supports tumor survival under therapeutic pressure. Targeting these metabolic vulnerabilities—such as glutamine dependence, *FSP1*-mediated ferroptosis defense, or mitochondrial plasticity—represents a promising avenue to enhance cisplatin efficacy and counteract resistance (**Figure 2**).

### 3.3 Evasion of regulated cell death

The efficacy of cisplatin in OSCC is, to a certain extent, dependent on the capacity of this platinum agent to induce regulated cell death. However, resistant cells can evade this fate through various mechanisms, with the regulation of death pathways such as apoptosis, pyroptosis, and autophagy constituting a key aspect (Dai et al., 2024).

Inhibition of the classical apoptotic pathway is a central mechanism in the development of resistance. Evidence from cisplatin-resistant OSCC cell models indicates that the expression of inhibitor of apoptosis proteins (IAPs), such as *XIAP* and *cIAP1*, is significantly upregulated in cisplatin-resistant cells, suppressing apoptosis by blocking caspase activity. Conversely, small-molecule IAP inhibitors (e.g., BV6 and Embelin) can restore cisplatin sensitivity in these resistant cells (Gao et al., 2024). The activity of key apoptotic executioners, *caspase-3* and *caspase-9*, is also attenuated in resistant cells (Su et al., 2020). Furthermore, overexpression of the transcription factor FOXM1 has been shown to inhibit apoptosis induced by paclitaxel in cisplatin-resistant cells, leading to antagonistic effects in combination chemotherapy (Choi et al., 2020). TME also contributes to this regulation; mesenchymal stem cells can confer stronger cisplatin tolerance to OSCC cells by activating the *PDGFR-α/AKT* pathway, upregulating the anti-apoptotic protein Bcl-2, and reducing the pro-apoptotic protein Bid (Wang et al., 2020).

Beyond apoptosis, a study based on the TCGA database constructed a prognostic model using pyroptosis-related genes and found a significant correlation between the expression of pyroptosis regulators and cisplatin sensitivity in OSCC patients, suggesting pyroptosis as a potential biomarker for cisplatin efficacy (Zeng et al., 2022). In contrast, autophagic cell death plays a complex and context-dependent dual role in cisplatin resistance. In some contexts, cisplatin can simultaneously induce both apoptosis and autophagic cell death in OSCC cells, and early-stage inhibition of autophagic cell death did not significantly sensitize cells to cisplatin in this model, indicating that autophagic cell death may not be a crucial pro-survival mechanism in this context (Magnano et al., 2021). Conversely, in established resistance, aberrant activation of autophagic pathways can serve as a protective mechanism. For instance, the circular RNA *circAP1M2* is highly expressed in resistant cells and promotes resistance by activating autophagic pathways via the *miR-1249-3p-ATG9A* axis (Wenhao et al., 2023). In another example, the tumor-suppressive *circ-PKD2* enhances cisplatin-induced cell death by upregulating *ATG13* via sponging *miR-646*, thereby activating autophagic cell death (Gao et al., 2022). These observations highlight that the role of autophagic cell death in resistance is highly dependent on the specific molecular context, and precise modulation of this process represents a potential therapeutic direction.

In summary, cisplatin resistance in OSCC involves the suppression of apoptosis, context-dependent regulation of autophagic cell death, and interactions with immune checkpoint molecules. Future combined intervention strategies targeting these nodes—such as IAP inhibitors, specific autophagic cell death node modulators combined with *BRD4* or immune checkpoint inhibitors—hold promise for developing new comprehensive treatment approaches to overcome resistance. (**Figure 2**).

### 3.4 Synergistic drive of EMT and tumor stemness

The acquisition of cisplatin resistance in OSCC cells confers enhanced migratory and invasive capabilities and an enrichment of CSCs, a phenotype that drives tumor recurrence and metastasis (Shahoumi, 2021; Siqueira et al., 2023).

The increased migratory and invasive potential of cisplatin-resistant OSCC cells is closely linked to the activation of EMT, typically marked by downregulation of *E-cadherin* and upregulation of mesenchymal markers such as *N-cadherin*. This process is finely regulated by a multi-layered signaling network. First, cell membrane receptors and their downstream pathways are key drivers (Choi et al., 2019; de Morais et al., 2025). Activation of EGFR signaling, which can be blocked by the *EGFR* inhibitor cetuximab, reverses EMT and suppresses cell migration (Choi et al., 2021). The *TGF-β/Smad* pathway, negatively regulated by tumor-suppressive miRNAs such as *miR-132* and *miR-149-5p*, promotes cell motility and chemoresistance upon activation (Luo et al., 2019; Chen et al., 2020). Second, extrinsic factors within the TME also play important roles. For example, the oral pathogen *Porphyromonas gingivalis* can synergistically promote both cisplatin resistance and migratory ability in OSCC cells by activating the Wnt/NFAT signaling pathway, leading to downregulation of *p53* and *E-cadherin* (Da et al., 2021). Furthermore, factors secreted by tumor cells themselves can create a vicious cycle. Cancer stem-like cells with high *CD10* expression secrete *IL-8*, which in turn enhances *CD10*-positive cancer cell migration, invasion, and drug resistance in an autocrine/paracrine manner by activating the p-ERK signaling pathway (Pu et al., 2021). Targeted interventions against these nodes have shown potential. For instance, the natural product Dayicin effectively blocks EMT by inhibiting the expression of *MMP-2/MMP-9* and the activity of the MAPK (ERK1/2, p38) signaling pathway, thereby suppressing cancer cell migration and invasion and exerting synergistic effects with cisplatin (Kang et al., 2025).

Notably, the activation of EMT and the acquisition of stem-like properties are biologically interconnected and are coordinately driven by a core molecular network. Highly expressed core stem cell transcription factors (e.g., *Nanog*, *SOX2*) in resistant cells not only maintain self-renewal capacity but also directly regulate EMT-related genes such as *Slug*, thus functionally linking stemness with motility (Kashyap et al., 2020; Sacco et al., 2023). The expansion of the CSC subpopulation is a central driver of resistance. In resistant cells and three-dimensional tumor sphere models, CSC markers such as *CD44* and *Oct4* are significantly enriched and this enrichment correlates with enhanced cisplatin resistance (Ikeda-Motonakano et al., 2023). The underlying molecular mechanisms involve multi-layered, precise regulation. At the level of key protein drivers, keratin 17 (*KRT17*) directly enhances CSC properties by activating the integrin β4/α6–FAK/Src/ERK signaling axis and stabilizing β-catenin (Jang et al., 2022), while aberrantly high expression of Bruton’s tyrosine kinase (*BTK*) promotes the expression of stemness factors like *Nanog* and *CD133*, and *BTK* inhibition sensitizes cells to cisplatin (Liu et al., 2021). At the epigenetic and metabolic regulatory levels, histone deacetylase *HDAC6* accumulates in CSCs, maintaining stemness by alleviating oxidative stress and DNA damage (Tavares et al., 2022); the histone methyltransferase *EZH2* promotes CSC accumulation by silencing the *APC* gene and activating the Wnt/β-catenin pathway (Milan et al., 2023); and the ferroptosis suppressor protein *FSP1* protects CSCs from death by regulating lipid metabolism and iron homeostasis (Wu et al., 2024). At the microenvironmental signaling level, inflammatory factors (e.g., *TNF-α*) derived from tumor-associated macrophages further promote CSC expansion and survival by activating signaling axes such as *NF-κB/SIRT1* or *NF-κB/LY6E* (de Castro et al., 2024; Hu et al., 2025).

In summary, cisplatin-resistant OSCC cells, through the synergistic acquisition of EMT and CSC traits, form cellular subpopulations with strong motility, self-renewal capacity, and therapy resistance. This multifaceted process is coordinately regulated by a multidimensional signaling network, providing multiple layers of potential targets for therapeutic intervention. (**Figure 2****).**

### 3.5 TME and immune cell infiltration

Cisplatin resistance arises not solely from intrinsic tumor cell alterations but is also closely linked to the systemic remodeling of the TME. Through immunosuppression, metabolic adaptation, and intricate intercellular communication, the TME collaboratively establishes a niche that supports tumor survival and drug resistance (Chen and Chang, 2019).

First, an immunosuppressive microenvironment is pivotal in driving resistance. The composition and functional states of immune cells within the TME dynamically change, profoundly influencing therapeutic outcomes. Immunohistochemical analysis of post-treatment specimens from patients with locally advanced OSCC (primarily stage III/IV) has shown that after neoadjuvant chemoradiotherapy, macrophages in tumor tissues tend to polarize towards the M2 phenotype (anti-inflammatory/pro-tumorigenic), a state associated with immunosuppression and poor prognosis (Weber et al., 2024). Furthermore, the cytokine network within the TME (e.g., *TNF*, *TGF-β*) also participates in shaping this immunosuppressive milieu and activating resistance pathways in tumor cells (Wang et al., 2025). Second, hypoxia, a central feature of the solid tumor TME, triggers broad adaptive responses by stabilizing transcription factors such as *HIF-1α*. Hypoxia can induce the upregulation of key glycolytic enzymes like *GAPDH*, which not only promotes tumor cell migration, invasion, and cisplatin resistance but is also linked to immunosuppressive states such as reduced CD8^+^ T cell infiltration, thereby tightly coupling metabolic reprogramming, malignant phenotypes, and immune evasion (Peng et al., 2023). Concurrently, hypoxic conditions can stimulate tumor cells to secrete *IL-8*, which synergistically enhances EMT, migratory/invasive capacities, and chemoresistance by activating the *NF-κB* signaling pathway (Joshi et al., 2023). Moreover, the active regulation by stromal cells serves as a crucial conduit for transmitting resistance signals. Cancer-associated fibroblast (CAFs) exhibit functional heterogeneity, wherein pro-tumorigenic CAFs (CAF-P) can actively confer cisplatin resistance to tumor cells by delivering factors such as *miR-876-3p* via exosomes, thereby suppressing the expression of tumor suppressor genes like *IGFBP3* in recipient cells (Kang et al., 2024). Finally, immune checkpoint molecules within the TME are direct mediators of immune escape. Notably, cisplatin treatment itself may induce the expression of immunosuppressive molecules such as *PD-L2* (Sudo et al., 2020). This observation suggests that combining cisplatin with immune checkpoint inhibitors (e.g., anti-PD-1 antibodies) can synergistically enhance anti-tumor immunity by reversing the immunosuppressive state of the TME, an approach that has shown potential to overcome resistance in both preclinical and clinical studies (Jang et al., 2024; An et al., 2025).

In conclusion, cisplatin resistance is an adaptive process predominantly orchestrated by the TME through multifactorial interactions. The imbalance in immune cell polarization, hypoxia-induced metabolic remodeling, exosome-mediated stromal-tumor communication, and upregulation of immune checkpoint signals collectively form a dynamic and protective microenvironmental network. Therefore, interventional strategies targeting the TME—such as modulating macrophage polarization, inhibiting hypoxia signaling axes, blocking detrimental stromal-tumor crosstalk, or combining therapies with immune checkpoint inhibitors—represent key directions for overcoming cisplatin resistance and improving therapeutic responses. (**Figure 2****).**

## 4 Strategies to overcome cisplatin resistance

### 4.1 Multi-target combination therapy

Based on the mechanistic understanding of cisplatin resistance in OSCC, combination strategies have emerged that target key nodes, including cell cycle checkpoints (e.g., the *MASTL* inhibitor GKI-1 (Gouttia et al., 2022), autophagic pathways (e.g., chloroquine (Li et al., 2020), and epigenetic regulators (e.g., the *HDAC6* inhibitor Tubastatin A (Tavares et al., 2022) and *EZH2* inhibitors (Milan et al., 2023). These approaches aim to restore tumor cell sensitivity to cisplatin by targeting key molecular pathways, modulating cellular homeostasis, and reversing epigenetic abnormalities.

First, small-molecule inhibitors targeting critical resistance-associated proteins represent one of the most direct strategies. For instance, IAPs (e.g., *XIAP*), which are highly expressed in resistant cells, can be targeted by small-molecule inhibitors such as BV6 and Embelin (Gao et al., 2024). These agents synergistically enhance cisplatin-induced apoptosis and reverse drug resistance (Gao et al., 2024). Another important target involves the cell cycle checkpoints and DNA damage repair machinery (Gouttia et al., 2022). The cell cycle kinase *MASTL*, whose upregulation promotes resistance via the *ENSA*-PP2A/B55 axis, can be inhibited by the specific inhibitor GKI-1 (Gouttia et al., 2022). At doses that do not interfere with mitosis, GKI-1 effectively enhances cisplatin-induced DNA damage and apoptosis in OSCC cell lines, demonstrating promising synergistic antitumor effects in xenograft mouse models (Gouttia et al., 2022).

Beyond directly targeting driver proteins, precise modulation of autophagy provides another effective route to reverse cisplatin resistance. For example, nanoparticles co-delivering cisplatin and the autophagy inhibitor chloroquine can effectively block autophagic flux, elevate reactive oxygen species levels, and promote apoptosis, thereby overcoming resistance (Li et al., 2020). The underlying molecular mechanisms involve the regulation of autophagy-related proteins such as *ATG12* and *LC3B-II* (Su et al., 2025). Conversely, in certain contexts, modulating autophagy can also synergize with chemotherapy. For instance, FTY720, a sphingosine kinase 1 antagonist, has been shown to induce autophagosome accumulation and enhance radiation-induced apoptosis in breast cancer cells, potentially by disrupting autophagic flux and shifting the cell death response toward apoptosis (Marvaso et al., 2014). When combined with paclitaxel, this agent cooperatively inhibits tumor growth and stem-like properties (Torres et al., 2024). Furthermore, non-coding RNA networks intricately regulate autophagy and resistance. For example, *circAP1M2* induces autophagy-associated resistance through the *miR-1249-3p-ATG9A* axis (Wenhao et al., 2023), whereas exosome-delivered *miR-30a* can restore cisplatin sensitivity by targeting *Beclin1* and *Bcl-2* (Kulkarni et al., 2020). These findings underscore that interventions targeting the autophagy pathway must be based on a thorough understanding of the specific resistance context.

Targeting epigenetic mechanisms that regulate gene expression represents a profound strategy to reverse cisplatin resistance. Epigenetic agents remodel the chromatin state and transcriptional programs of resistant cells by intervening in DNA methylation, histone modifications, and RNA methylation, thereby influencing multiple processes such as DNA damage repair, apoptosis, metabolism, and CSC properties (Lumpp et al., 2024; Yu et al., 2025). One core approach involves directly targeting chromatin-modifying enzymes, including histone deacetylase (HDAC) inhibitors such as Tubastatin A (targeting *HDAC6*) or compounds containing hydroxamate structures, which increase histone acetylation, induce oxidative stress and DNA damage, and selectively eliminate resistant cells and CSCs (Tavares et al., 2022; Aleksandrova et al., 2023). Another target is the histone methyltransferase *EZH2*, which catalyzes the repressive mark H3K27me3; *EZH2* inhibitors can relieve the silencing of *APC*, a suppressor of the Wnt/β-catenin pathway, and when combined with β-catenin inhibitors, they synergistically reduce CSCs and restore cisplatin sensitivity (Milan et al., 2023). Additionally, DNA methyltransferase inhibitors (DNMTis) such as 5-aza-2’-deoxycytidine (5-aza-dC) can reverse hypermethylation at promoter regions of tumor suppressor genes like *HOXA5*, restore tumor suppressor expression, promote cell death, and thereby sensitize cells to cisplatin (Lima et al., 2022; Chen et al., 2025). Furthermore, inhibitors targeting the RNA m^6^ A methyltransferase *METTL3*—which stabilizes various pro-oncogenic mRNAs such as *c-Myc* and *PD-L1*—can suppress tumor progression and reverse chemotherapy resistance (Liu et al., 2025). Another strategic direction is to inhibit key signaling pathways closely coupled with epigenetic regulation. For example, *NF-κB* signaling is constitutively activated in resistant cells, and *NF-κB* inhibitors (e.g., CBL0137, emetine) not only block pro-survival signals but also downregulate the downstream deacetylase *SIRT1*, increase histone acetylation, effectively reduce CSCs, and reverse resistance, illustrating the interplay between signaling and epigenetic regulation (de Castro et al., 2024). Interventions targeting different layers of epigenetic regulators—such as DNMTs, HDACs, *EZH2*, and *METTL3*—or key interacting pathways like *NF-κB* can systematically reshape the gene expression profile of resistant cells, offering multifaceted avenues for drug development to overcome cisplatin resistance.

**Figure 3:**
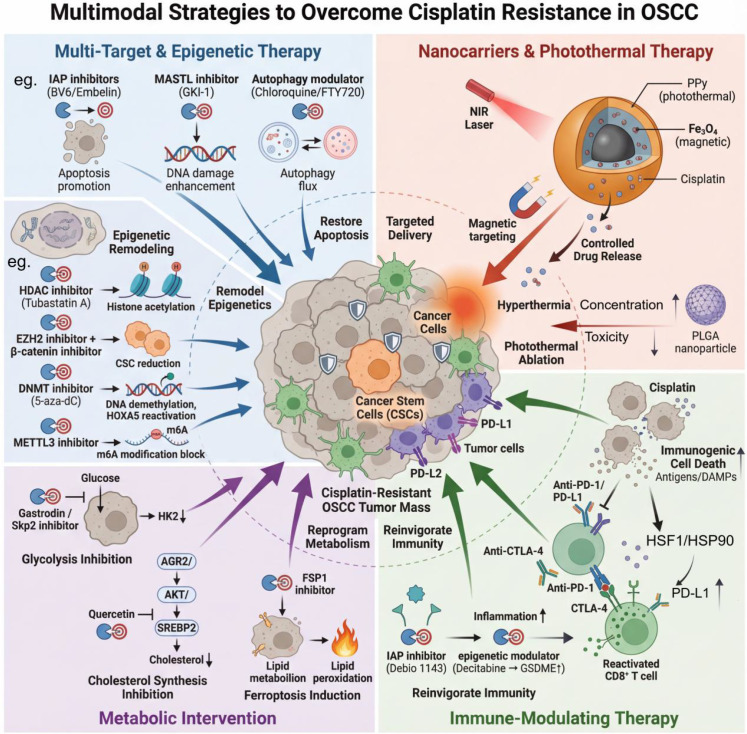
Multimodal strategies to overcome cisplatin resistance in OSCC. This schematic outlines four integrated therapeutic approaches targeting resistant OSCC tumors, which comprise cancer cells, CSCs, and immune checkpoint molecules (PD-L1, PD-L2). Multi-target and epigenetic therapies employ small-molecule inhibitors against key resistance nodes: IAP inhibitors (BV6/Embelin) promote apoptosis, MASTL inhibitor (GKI-1) enhances DNA damage, and autophagy modulators (chloroquine/FTY720) regulate autophagic flux. Epigenetic remodeling involves HDAC inhibitors (Tubastatin A), combined EZH2/β-catenin inhibitors to reduce CSCs, DNMT inhibitors (5-aza-dC) to reactivate HOXA5, and METTL3 inhibitors to block m⁶A modification. Nanocarrier-based approaches utilize multifunctional nanoparticles (e.g., PLGA nanoparticles, CDDP@PPy@Fe₃O₄) that integrate photothermal conversion via polypyrrole (PPy), magnetic targeting via Fe₃O₄, and controlled cisplatin release, enabling near-infrared (NIR)-triggered hyperthermia ablation and enhanced tumor accumulation with reduced systemic toxicity. Immune-modulating strategies leverage cisplatin-induced immunogenic cell death, which releases antigens/DAMPs and upregulates PD-L1 via HSF1/HSP90. Immune checkpoint inhibitors (anti-PD-1/PD-L1, anti-CTLA-4) reinvigorate CD8⁺ T cells, while combinations such as IAP inhibitors (Debio 1143) plus epigenetic modulators (decitabine inducing GSDME) enhance inflammation and immune infiltration. Metabolic interventions target glycolysis via gastrodin/Skp2 inhibitor (suppressing HK2), cholesterol synthesis via quercetin (targeting AGR2/AKT/SREBP2), and FSP1 inhibition to disrupt lipid metabolism and induce ferroptosis through lipid peroxidation. Collectively, these multimodal strategies reprogram metabolism, remodel epigenetics, enhance immunogenicity, and improve drug delivery to overcome cisplatin resistance in OSCC.

In summary, strategies to overcome cisplatin resistance in OSCC have evolved into a multi-layered interventional framework: from small-molecule inhibitors targeting key resistance proteins such as *MASTL*, to precise modulators of autophagic homeostasis, and further to agents that remodel the tumor epigenetic landscape, including HDAC inhibitors, DNMT inhibitors, and m^6^ A modification inhibitors. Through distinct mechanisms, these strategies collectively aim to restore cellular sensitivity to DNA damage, trigger apoptosis, and eliminate resistant stem-like subpopulations. Future efforts should be based on the precise identification of patient-specific molecular subtypes of resistance, the selection or combination of the above strategies is expected to effectively break through the bottleneck of cisplatin resistance in clinical practice. (**Figure 3**).

### 4.2 Nanodrug carriers and photothermal combination therapy

The application of nanotechnology in the treatment of OSCC offers innovative strategies to optimize the delivery of chemotherapeutic agents such as cisplatin (Liu et al., 2025). These approaches center on enhancing targeting precision, reducing systemic toxicity, and enabling synergistic combination with PTT to improve efficacy (Liu et al., 2025).

A representative example is the CDDP@PPy@Fe_3_O_4_ magnetic nanoplatform, a composite in which cisplatin is loaded onto polypyrrole-coated iron oxide nanoparticles (Liu et al., 2025). The design of this platform integrates magnetic targeting with photothermal function: under near-infrared laser irradiation, PPy generates localized hyperthermia to ablate tumor cells, while Fe_3_O_4_ enhances accumulation at the tumor site. This configuration achieves a synergistic combination of chemotherapy and PTT, demonstrating potent antitumor effects and favorable biocompatibility both *in vitro* and *in vivo* (Liu et al., 2025). This example underscores the advantage of nanocarriers in enabling precise combination therapies.

The fundamental strength of nanocarriers lies in their ability to reshape the *in vivo* distribution of drugs. For instance, PRV111—a transmucosal patch delivering cisplatin-loaded chitosan nanoparticles—has demonstrated significant antitumor efficacy in OSCC (Goldberg et al., 2022). Preclinically, this system achieved high local drug retention with minimal systemic exposure, leading to robust tumor regression. In a phase I/II trial, neoadjuvant PRV111 application resulted in approximately 69% tumor volume reduction within seven days and an overall response rate exceeding 87%, with no dose-limiting toxicities or systemic adverse events. Cisplatin concentrations in tumor tissues were markedly higher than those achieved with intravenous administration, while systemic levels remained negligible, effectively abrogating typical cisplatin-induced toxicities (Goldberg et al., 2022).

Similarly, biodegradable polymeric nanoparticles such as those made from poly(lactic-co-glycolic acid) (PLGA) can markedly increase intratumoral drug concentration and alleviate systemic toxicity (Moreno et al., 2010). Combining chemotherapy with PTT further amplifies the therapeutic potential of nanoplatforms. Beyond the aforementioned magnetic system, multifunctional nanosystems based on graphene oxide have been developed, modified with targeting molecules (e.g., FAP-targeting peptides) and loaded with chemotherapeutic drugs like doxorubicin. These platforms enable targeted and pH-responsive drug release while generating a potent photothermal effect upon near-infrared irradiation, directly ablating tumor cells and promoting local drug release. This combined chemo-photothermal strategy overcomes the limitations of monotherapies, demonstrating synergistic antitumor efficacy in OSCC (Li et al., 2023).

In summary, multifunctional nanoplatforms represented by CDDP@PPy@Fe_3_O_4_ provide a multimodal synergistic solution to address the challenges of resistance and toxicity in cisplatin-based OSCC treatment. By integrating targeted delivery, controlled drug release, and PPT, these systems highlight the significant potential of nanomedicine in achieving precise, effective, and low-toxicity treatments. (**Figure 3**).

### 4.3 Immune-modulating combination therapies

Cisplatin has demonstrated enhanced efficacy in OSCC and HNSCC when combined with immune checkpoint inhibitors (ICIs), establishing this approach as a key therapeutic strategy.

ICIs, which restore T-cell function by blocking immunosuppressive signals such as *PD-1/PD-L1*, have demonstrated clinical benefits in advanced-stage patients (Dunn et al., 2025). Cisplatin contributes to this combination by synergizing with other antitumor agents such as cetuximab (as in the EXTREME regimen) to induce immunogenic cell death, which releases tumor antigens and damage-associated molecular patterns, thereby activating immune responses within the TME and laying the groundwork for synergistic therapy (De Azevedo et al., 2022). At the molecular level, cisplatin can modulate critical immune checkpoint molecules. *In vitro* studies in OSCC cell lines reveal that this platinum agent upregulates *PD-L1* expression on tumor cells via activation of the *HSF1-HSP90* axis. While this upregulation may initially suppress local T-cell activity, the finding also underscores the potential rationale for combining cisplatin with *PD-1/PD-L1* inhibitors (Sasaya et al., 2023).

Furthermore, emerging clinical evidence demonstrates that such combinations can favorably remodel the tumor immune microenvironment. For instance, when neoadjuvant chemotherapy is combined with PD-1 inhibitors, increased infiltration of M1-type (pro-inflammatory/anti-tumor) macrophages directly correlates with significantly improved pathological response rates, highlighting the decisive role of immune cell phenotypes in treatment efficacy (He et al., 2025). Inducing the expression of the pyroptosis-executing protein *GSDME* using epigenetic modulators such as decitabine can trigger robust inflammatory responses, reverse the immunosuppressive microenvironment, and enhance the synergy between chemotherapy and immunotherapy (Zi et al., 2023).

The clinical promise of this combination is increasingly being validated. For instance, a short-course induction regimen of cisplatin/docetaxel combined with the *PD-L1* inhibitor durvalumab and the *CTLA-4* inhibitor tremelimumab achieved a high rate of pathological complete response in patients with locally advanced HNC, accompanied by a significant increase in intratumoral CD8^+^ T-cell infiltration (Hecht et al., 2020; Hecht et al., 2022). Additionally, combining IAP inhibitors (e.g., Debio 1143) with cisplatin has been shown to promote the infiltration of immune cells, including CD8^+^ T cells, within tumors and upregulate PD-1/PD-L1 expression, suggesting promise for further combination with immune checkpoint inhibitors (Gomez-Roca et al., 2022).

In summary, the combination of cisplatin with immunotherapy provides a novel direction to overcome therapeutic limitations in OSCC/HNSCC through multiple mechanisms: induction of immunogenic cell death, regulation of immune checkpoint molecules, and remodeling of the immune microenvironment. Future large-scale randomized trials are warranted to optimize combination protocols, identify the patient populations most likely to benefit, and determine the optimal sequencing of therapies. (**Figure 3**).

### 4.4 Metabolic intervention strategies

Cisplatin resistance is closely linked to metabolic reprogramming in tumor cells. Targeting these pathways—including enhanced glycolysis, altered cholesterol metabolism, and evasion of ferroptosis—targeting these pathways (e.g., hexokinase 2 (*HK2)* (Xie et al., 2023), *SREBP2* (Wang et al., 2024), *FSP1* (Wu et al., 2024) represents a rational strategy to overcome resistance.

Regarding glucose metabolism, enhanced glycolysis is commonly observed in resistant cells, with the overexpression of *HK2* being a key driver. The natural compound gastrodin can downregulate *HK2* expression by inhibiting the *Skp2−Akt* signaling axis, thereby suppressing glycolysis and restoring cisplatin sensitivity in resistant cells. This effect is further enhanced when combined with a *Skp2* inhibitor (Xie et al., 2023). Abnormalities in lipid metabolism also profoundly influence resistance. Dysregulated cholesterol metabolism is a common feature; the flavonoid quercetin can sensitize cells to cisplatin by inhibiting the *AGR2/AKT/SREBP2* axis and downregulating key genes involved in cholesterol synthesis (Wang et al., 2024). Additionally, upregulation of the mitochondrial−specific lipid cardiolipin is associated with cellular resistance to cisplatin−induced apoptosis (Thorne et al., 2021).

Notably, metabolic reprogramming is tightly connected to ferroptosis, The ferroptosis suppressor protein *FSP1* is highly expressed in tissues from recurrent patients and in drug−tolerant persister cells. By modulating lipid metabolism and intracellular iron homeostasis, *FSP1* protects CSC-like cells from ferroptosis, thereby maintaining the resistant phenotype. Targeting *FSP1* induces ferroptosis and reverses resistance (Wu et al., 2024). This observation indicates that interfering with specific metabolic vulnerabilities—such as *FSP1*−mediated resistance to lipid peroxidation—represents a promising new direction for overcoming cisplatin resistance.

In summary, pharmacologically targeting hyperactive glycolysis and cholesterol metabolism or exploiting metabolic dependencies to induce ferroptosis can reshape the fate of resistant cells at the metabolic level. These approaches provide multi−faceted combination strategies to overcome cisplatin resistance in OSCC. (**Figure 3****).**

## 5 Clinical challenges and future research directions in cisplatin resistance

### 5.1 Developing predictive models and translating biomarkers into clinical practice

To address the clinical challenges posed by cisplatin resistance in OSCC, researchers are focusing on two key areas: developing non-invasive/minimally invasive biomarker detection techniques for early diagnosis and dynamic monitoring, and constructing multi-omics-based predictive models for precise intervention. These complementary efforts collectively advance the goal of personalized treatment strategies.

Early diagnosis and dynamic monitoring rely on identifying reliable resistance-associated molecules in clinical samples, for which non-invasive liquid biopsy provides a key platform (Adhit et al., 2023; Ma et al., 2023). For instance, circulating tumor cells (CTCs) and exosomes have emerged as sensitive indicators for tracking resistance dynamics. Antibody-functionalized materials can efficiently capture and culture CTCs for subsequent drug sensitivity analysis (Arora et al., 2022), while specific miRNAs (e.g., *miR-30a*) or proteins carried by exosomes can directly reflect tumor resistant status (Kulkarni et al., 2020). Saliva, as a local secretion in OSCC, also serves as an ideal source for detecting resistance-related markers. These technologies make real-time monitoring during therapy feasible.

The core of translating these molecular discoveries into clinical decision-making tools lies in constructing systematic predictive models. By utilizing public databases such as TCGA and GEO and integrating multi-omics data (e.g., transcriptomics, epigenomics) through machine learning algorithms (e.g., LASSO regression, random survival forest), it is possible to identify gene signatures (e.g., *STC2, NDRG1*) closely associated with resistance and prognosis. These models can be used to develop risk scores or nomograms for quantifying patient risk and therapeutic response (Lu et al., 2024; Liu et al., 2025). Such predictive tools not only independently predict survival but also reveal differences in the immune microenvironment (e.g., M2 macrophage infiltration) and drug sensitivity across different risk groups, thereby informing treatment choices (Luo et al., 2023).

Underpinning these detection techniques and predictive models is a series of validated, multi-level key biomarkers, which fall into two main categories. The first comprises protein-coding genes, such as the tumor suppressor *Naa10p* and *MTMR6*, and pro-resistance factors like *RRBP1* (Shriwas et al., 2021; Sun et al., 2021; Lee et al., 2025), Expression levels of these genes are specifically altered in resistant cells and tissues and are directly correlated with patient treatment response. The second category consists of non-coding RNAs, including miRNAs (e.g., serum-elevated *miR-92b-3p*) and lncRNAs (e.g., *SNHG1, STARD4-AS1*). These molecules are not only stably present in body fluids, facilitating non-invasive detection, but also directly involved in resistance formation by regulating key pathways such as Notch and ferroptosis, thereby serving dual roles in prediction and mechanistic elucidation (Wang et al., 2020; Li et al., 2024; Piao et al., 2025).

**Figure 4:**
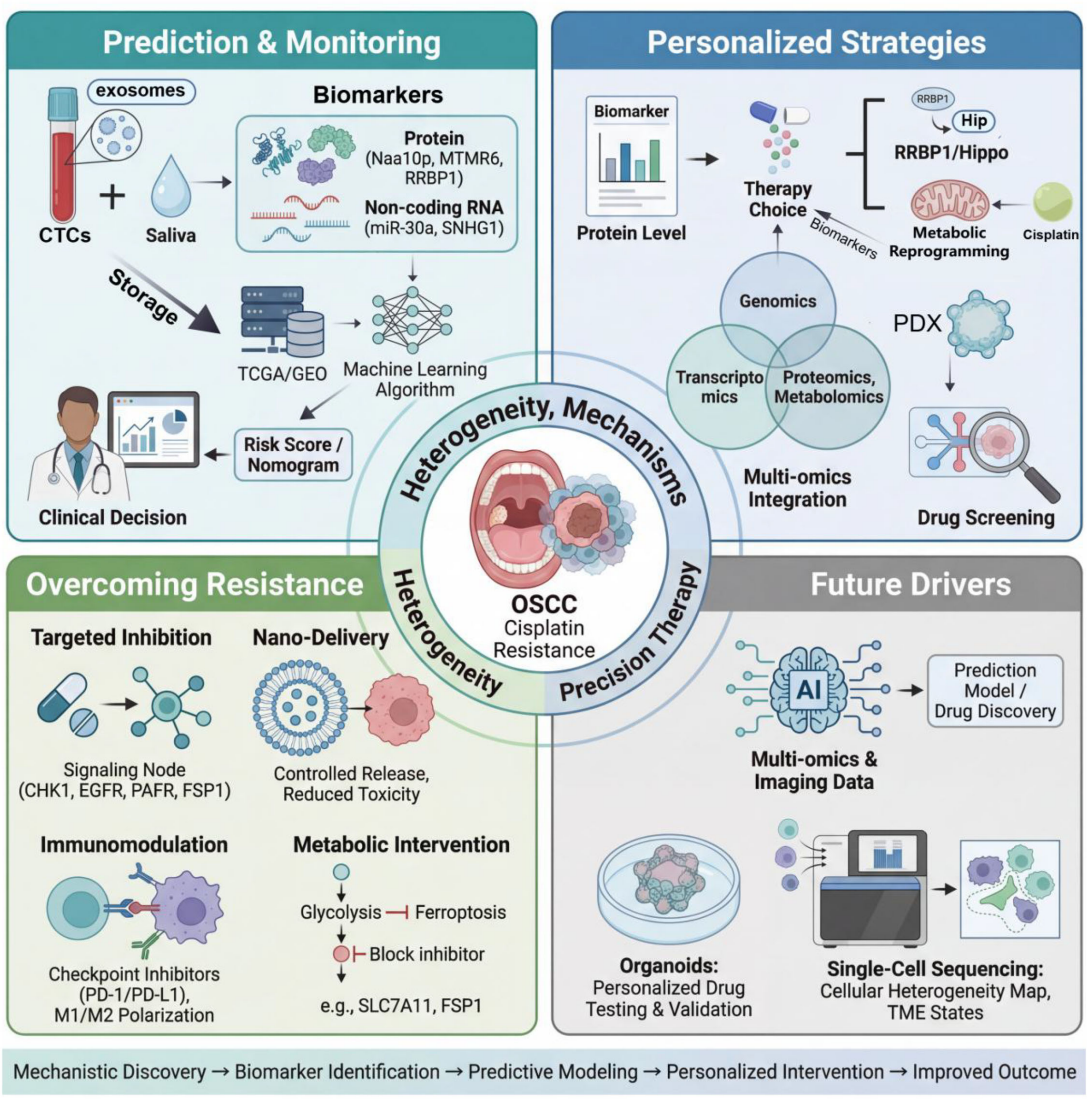
A precision therapy paradigm for overcoming cisplatin resistance in OSCC: from mechanistic discovery to personalized intervention. This schematic illustrates a comprehensive precision medicine framework addressing cisplatin resistance in OSCC, organized around tumor heterogeneity and underlying mechanisms. The workflow progresses from prediction and monitoring to personalized strategies, resistance reversal, and future technological drivers. Non-invasive liquid biopsy utilizing CTCs, exosomes, and saliva enables detection of protein (Naa10p, MTMR6, RRBP1) and non-coding RNA biomarkers (miR-30a, SNHG1), with data deposited in TCGA/GEO databases to support machine learning-based risk stratification and clinical decision-making. Personalized strategies integrate multi-omics profiling (genomics, transcriptomics, proteomics, metabolomics) with patient-derived xenograft (PDX) drug screening to tailor interventions targeting specific mechanisms such as RRBP1/Hippo pathway dysregulation or metabolic reprogramming. Approaches to overcome resistance encompass four pillars: targeted inhibition of signaling nodes (CHK1, EGFR, PAFR, FSP1); nano-delivery systems for controlled release and reduced toxicity; immunomodulation via checkpoint inhibitors (PD-1/PD-L1) and macrophage polarization; and metabolic intervention targeting glycolysis and ferroptosis (SLC7A11, FSP1 inhibitors). Emerging future drivers include artificial intelligence integrating multi-omics and imaging data for predictive modeling and drug discovery, patient-derived organoids for personalized drug testing, and single-cell sequencing to map cellular heterogeneity, TME states, and DTPs. Collectively, this framework delineates a translational workflow from mechanistic discovery and biomarker identification through predictive modeling to personalized intervention, ultimately aiming to improve clinical outcomes in cisplatin-resistant OSCC.

In summary, integrating dynamic monitoring based on liquid biopsy with systematic predictive models derived from multi-omics, and grounding these approaches in functionally validated multi-level biomarkers, forms a comprehensive framework for early warning, efficacy evaluation, and mechanism exploration. This integrated approach establishes a solid foundation for ultimately achieving precise and individualized management of cisplatin therapy in OSCC. (**Figure 4****).**

### 5.2 Formulating individualized treatment strategies

The formulation of individualized treatment strategies is crucial in OSCC, particularly given the significant variability in cisplatin efficacy. Predicting treatment response based on molecular biomarkers provides a foundation for precision medicine. Studies have shown that the *Naa10p* protein is highly expressed in the saliva of cisplatin-sensitive patients, and *Naa10p* overexpression can enhance cisplatin sensitivity (Sun et al., 2021). Low expression of *MTMR6* is associated with cisplatin resistance and poor prognosis and is regulated by *miR-544a*, suggesting that both could serve as predictive markers (Lee et al., 2025). *CAV1* reduces cisplatin sensitivity by inhibiting ferroptosis, and its elevated levels predicts unfavorable outcomes (Zhang et al., 2025). Detecting these molecular markers can help guide clinical treatment decisions.

Integrating multi-omics data further enhances the systematic nature of clinical decision-making. By combining genomic, transcriptomic, proteomic, and metabolomic data, the mechanisms underlying cisplatin resistance can be systematically elucidated. For example, *RRBP1* drives resistance by regulating the Hippo pathway effector *YAP1*, and targeting *RRBP1* can restore cisplatin sensitivity (Shriwas et al., 2021). Metabolomic analyses have revealed that OSCC cells undergo specific metabolic reprogramming following cisplatin exposure, and the associated metabolites and pathways can serve as early-response biomarkers (Chen et al., 2022). Moreover, patient-derived models (e.g., PDX) combined with microfluidic chip technology and multi-omics data can simulate tumor heterogeneity and facilitate drug screening, thereby advancing the optimization of treatment regimens (Mehta et al., 2024; Feng et al., 2025).

In summary, biomarker-guided prediction and multi-omics integration are key to advancing individualized cisplatin therapy in OSCC. Future efforts should focus on deepening the integration of multi-omics data with clinical information, developing efficient detection technologies, and constructing data-driven decision-support systems, ultimately enabling more precise and effective treatment for OSCC. (**Figure 4****).**

### 5.3 Comprehensive strategies to overcome cisplatin resistance in OSCC: from molecular targets to technological applications

As detailed in section 4, a diverse arsenal of interventions has been developed to counter cisplatin resistance in OSCC, including small-molecule inhibitors targeting specific resistance nodes, nanotechnology-based drug delivery systems, immunomodulatory combinations, and metabolic interventions. While each approach has demonstrated promise in preclinical or early clinical settings, the central challenge now lies in integrating these strategies into coherent, clinically applicable treatment frameworks tailored to individual patient profiles.

The selection of optimal combination strategies should be guided by the dominant resistance mechanisms operative in each patient. High-throughput screening and functional genomics in cancer cell line panels have identified a portfolio of actionable targets that can inform this selection. For instance, *STK19* inhibition depletes the DNA repair enzyme *MGMT*, creating synthetic lethality with cisplatin in tumors harboring intact *MGMT* expression (Li et al., 2025). The small-molecule inhibitor TC-I 15 targets the CSC-associated integrin *ITGA2*, blocking collagen interaction and reversing stem-like phenotypes (Qi et al., 2025). Other emerging targets include the lncRNA *PVT1* driving resistance via the *miR-194-5p/HIF1α* axis, signaling nodes such as *CHK1/PI3K*, *EGFR*, and *PAFR*, and ferroptosis regulators including *ACACA* and *FSP1* (Wang et al., 2020; Yang et al., 2020; Kawasaki et al., 2021; Kanemaru et al., 2022; Wu et al., 2024; Xu and Jia, 2025). The clinical deployment of inhibitors against these targets will require companion diagnostic assays to identify patients most likely to benefit.

The true potential of multi-strategy synergy lies not in simply adding modalities, but in rationally combining them based on mechanistic complementarity and temporal dynamics. Key considerations include therapeutic sequencing, dose optimization, and toxicity management. Regarding sequencing, preclinical evidence suggests that priming the immune microenvironment with cisplatin before checkpoint blockade may optimize CD8^+^ T cell infiltration, raising the question of whether immunotherapy should be administered concurrently or following chemotherapy-induced immunogenic cell death (An et al., 2025). In terms of dose optimization, when combining cisplatin with targeted agents or nanocarriers, the optimal dose of each component may differ from monotherapy regimens. For example, the *MASTL* inhibitor GKI-1 enhances cisplatin-induced DNA damage at doses that do not disrupt normal mitosis, illustrating the potential for dose-sparing effects (Gouttia et al., 2022). Managing overlapping toxicities also requires careful consideration; nanocarrier formulations such as CDDP@PPy@Fe_3_O_4_ magnetic nanoplatforms can reduce systemic cisplatin exposure while maintaining intratumoral efficacy (Liu et al., 2025), but combining these platforms with immunotherapies or metabolic modulators necessitates rigorous safety evaluation.

Given the evolving nature of resistance, static pretreatment stratification is insufficient; however, emerging technologies now enable dynamic treatment adjustment. Liquid biopsy monitoring of circulating tumor cells, exosomal miRNAs such as *miR-30a*, or resistance-associated proteins can detect emerging resistance before clinical progression (Kulkarni et al., 2020). Molecular imaging techniques may visualize the activity of key resistance pathways, including *HIF-1α* under hypoxic conditions, in real time to guide timely intervention. Furthermore, adaptive trial designs that modify treatment based on early molecular response represent a promising paradigm for overcoming the adaptive plasticity of resistant tumors.

Ultimately, overcoming cisplatin resistance will require a platform that integrates multi-omics profiling to characterize the resistance landscape in each patient, functional testing using patient-derived organoids to validate drug sensitivity *in vitro (*Driehuis et al., 2019; Wang et al., 2025c; Zhang et al., 2025), and AI-powered decision support that integrates molecular data with clinical parameters to recommend optimal combination regimens and predict response dynamics (Keramida et al., 2025; Wan et al., 2025; Wang et al., 2025b). In summary, while the individual strategies to reverse cisplatin resistance have expanded dramatically, the next frontier lies in clinical integration. Future research must move beyond proof-of-concept studies to establish evidence-based frameworks for patient selection, rational combination design, dynamic monitoring, and adaptive therapy. Only through such systematic integration can the promise of multi-strategy synergy be translated into meaningful improvements in outcomes for OSCC patients (**Figure 4**).

### 5.4 Emerging technologies driving paradigm shifts in resistance research: artificial intelligence, organoids, and single-cell sequencing

With the exponential accumulation of multi-omics data and ongoing innovations in research methodologies, artificial intelligence (AI), organoid models, and single-cell sequencing have emerged as key drivers in deciphering the complexity of cisplatin resistance and advancing the implementation of precision therapy (Chen et al., 2022; Li et al., 2024; Xu et al., 2025). These technologies—spanning systemic prediction, functional simulation, and cellular-resolution analysis—provide unprecedented tools and perspectives for overcoming cisplatin resistance in OSCC.

Artificial intelligence and machine learning enable the integration of multimodal data—including genomic, transcriptomic, proteomic, metabolomic, and clinical imaging information—to construct high-accuracy predictive models for resistance and therapeutic decision-making (Chen et al., 2022; Liao et al., 2025). Deep-learning-based algorithms can automatically extract features of the TME from pathological slides to predict initial patient response to cisplatin and the risk of resistance (Li et al., 2025; Wang et al., 2025). Furthermore, AI-driven drug repurposing and virtual screening can identify potential small-molecule candidates capable of reversing resistance from vast compound libraries, significantly accelerating drug discovery. AI-based dynamic clinical decision-support systems hold promise for real-time optimization and adjustment of treatment regimens, moving toward truly data-driven individualized therapy (Keramida et al., 2025; Wan et al., 2025; Wang et al., 2025b).

Patient-derived organoid models offer an ideal platform for *in vitro* simulation of tumor heterogeneity, the microenvironment, and therapeutic response (Hill and D’Andrea, 2019). By culturing OSCC patient tissue into three-dimensional organoids, high-throughput cisplatin sensitivity testing and combination drug screening can be performed while preserving original tumor genetic and phenotypic characteristics (Driehuis et al., 2019; Wang et al., 2025c). This technology not only facilitates prospective prediction of patient-specific resistance patterns but also, when combined with gene-editing tools such as CRISPR-Cas9, enables functional validation of resistance targets in organoids, bridging the gap between “personalized drug sensitivity testing” and mechanistic investigation (Zhang et al., 2025).

Single-cell sequencing has fundamentally transformed our ability to understand cellular heterogeneity and the dynamic evolution of resistance. By performing single-cell analyses on sequential patient biopsies obtained before and after cisplatin treatment, or on sensitive and resistant tumor cell populations from model systems, rare subpopulations driving resistance (e.g., BASP1-positive resistant stem-like cells) can be identified (Li et al., 2024; Woo et al., 2025). This approach is particularly valuable for detecting and characterizing DTPs—the reversible, slow-cycling state that serves as a precursor to fully resistant clones. For example, single-cell analysis can reveal the distinct transcriptional signatures and metabolic states of DTPs, such as BASP1-positive persister stem-like cells, and elucidate the molecular switches governing the transition from the persister state to established resistance (Behera et al., 2026). The technology also characterizes shifts in immune cell states within the TME and reveals key molecular switches along the evolutionary trajectory of resistance (Nath and Bild, 2021; Cheng et al., 2025). For example, single-cell sequencing can precisely identify specific T-cell subsets mediating immune escape (Jerby-Arnon et al., 2018) or elucidate how intra-tumoral metabolic heterogeneity leads to differential drug responses, providing a direct rationale for developing therapies that target resistant subpopulations (Xu et al., 2023).

In summary, AI, organoids, and single-cell sequencing are complementary: single-cell technologies provide high-resolution mechanistic maps (Um et al., 2025), organoid models enable patient-specific functional validation and screening, and AI integrates multi-source data for systemic prediction and decision optimization. Systematically incorporating these cutting-edge technologies into OSCC cisplatin resistance research and clinical translation will significantly advance our understanding of resistance systems biology and ultimately catalyze a paradigm shift from “population-based treatment” to “precision intervention at the level of the individual patient—or even cellular subpopulation (Doerfler et al., 2025).” (**Figure 4****).**

## 6 Conclusion

Cisplatin remains a cornerstone in the treatment of OSCC, yet the clinical efficacy of this platinum agent is frequently compromised by the emergence of drug resistance. The present review synthesizes current evidence to demonstrate that cisplatin resistance is not attributable to a single cause, but rather constitutes a complex, adaptive system. This system arises from the interplay between tumor cell-intrinsic alterations and extrinsic microenvironmental factors (Cheng et al., 2021), encompassing multiple interconnected dimensions such as genetic and epigenetic reprogramming, metabolic remodeling, evasion of regulated cell death, acquisition of stem-like properties, and dysregulation of the immune landscape.

To address this multifaceted challenge, the research paradigm is shifting from purely mechanistic dissection toward the development of multi-target and multimodal intervention strategies. These strategies include the design of specific inhibitors against key resistance nodes, the use of nanotechnology for targeted drug delivery (Morgovan et al., 2025), the combination of cisplatin with immunotherapy to remodel the immunosuppressive microenvironment (Shibata et al., 2025), the exploitation of tumor-specific metabolic vulnerabilities (Zou et al., 2025). Collectively, such approaches represent an integrated therapeutic network targeting resistance across molecular, cellular, and microenvironmental scales.

Ultimately, overcoming cisplatin resistance requires a fundamental evolution in therapeutic approach, driven by emerging technologies. Patient-derived organoids offer a physiologically relevant platform for *in vitro* drug testing and personalized strategy validation (Um et al., 2025). Single-cell multi-omics technologies decode tumor heterogeneity at unprecedented resolution, pinpointing rare resistant subpopulations and the crosstalk of these populations with the microenvironment (Doerfler et al., 2025). Artificial intelligence and machine learning integrate vast multi-scale datasets to build predictive models and accelerate biomarker and drug discovery (Liu et al., 2025). The synergy of these technologies—where single-cell analysis provides high-resolution maps, organoids enable functional testing (Han et al., 2025), and AI facilitates data integration and optimization—is propelling the field from population-level observations toward precision interventions guided by cellular atlases and individualized models.

In summary, advancing against cisplatin resistance in OSCC necessitates a cohesive framework that links systematic mechanistic understanding, innovative therapeutic strategies, and cutting-edge translational technologies. Future efforts must focus on deepening systems-level comprehension of resistance dynamics and accelerating the clinical integration of organoid models, single-cell technologies, and AI-driven tools to enable data-informed, personalized clinical decision-making. This interdisciplinary, integrative path forward is essential to transition from standardized chemotherapy to truly tailored precision medicine, with the goal of significantly improving outcomes for patients with OSCC.

## 7 Limitations

Given the broad and integrative scope of this narrative review, we conducted a comprehensive literature search to synthesize the multifaceted mechanisms of and strategies against cisplatin resistance in oral squamous cell carcinoma (OSCC). We performed literature searches in the PubMed and Web of Science databases up to December 2025.

The search strategy employed a combination of keywords and Boolean operators, including terms such as: (“oral squamous cell carcinoma” OR “OSCC” OR “head and neck squamous cell carcinoma” OR “HNSCC”) AND (“cisplatin” OR “CDDP”) AND (“drug resistance” OR “chemoresistance”) AND (“therapeutic strategies” OR “reversal” OR “nanotechnology” OR “immunotherapy” OR “metabolic intervention” OR “emerging technologies”). This broad string was designed to capture the maximum breadth of relevant literature across the various topics discussed in this review.

Articles were selected based on their relevance to the thematic sections of the review, including molecular mechanisms, clinical challenges, and emerging therapeutic modalities. Priority was given to peer-reviewed original research, authoritative reviews, and key clinical trials that have shaped the current understanding of the field. Additionally, the reference lists of retrieved articles were manually screened to identify further relevant publications and ensure a thorough synthesis of the evidence.

While this review has comprehensively synthesized recent advances in understanding cisplatin resistance and corresponding reversal strategies in OSCC, several limitations should be acknowledged to guide future research directions:

### 7.1 Scope of literature search

As a narrative review, this analysis does not claim to be a systematic review or meta-analysis. The search, while comprehensive, primarily relied on two major English-language databases. Consequently, the review may not fully encompass every relevant study, particularly those published in non-English journals or regional publications, introducing potential language and geographic bias.

### 7.2 Heterogeneity and complexity of mechanistic studies

Cisplatin resistance involves multidimensional molecular networks and dynamic microenvironmental interactions (Zhou et al., 2025). Most current insights are derived from cell line or animal models, and the generalizability of these findings to human tumors requires validation in large-scale clinical cohorts (Niehr et al., 2018). Furthermore, the influence of OSCC subtypes, disease stages, and inter-patient heterogeneity on resistance mechanisms remains incompletely defined.

### 7.3 Challenges in clinical translation

Many promising reversal strategies discussed herein—including nano-targeted delivery, immunochemotherapy combinations, and metabolic interventions—remain in preclinical or early-phase clinical development. The long-term safety, efficacy, and potential for these approaches to elicit new forms of resistance require rigorous evaluation. Implementing personalized strategies also depends on robust molecular profiling and real-time monitoring, which currently face challenges related to technical standardization and cost-effectiveness in routine clinical practice (Aboaid et al., 2025; Herdiana, 2025).

### 7.4 Developmental stage of emerging technologies

Although organoid models, single-cell sequencing, and artificial intelligence hold tremendous promise, the application of these technologies in OSCC cisplatin resistance research remains nascent. Issues surrounding data interpretation, model generalizability, and seamless clinical integration necessitate further methodological refinement and multi-center validation.

### 7.5 Dynamic and adaptive nature of resistance

Current research often provides static snapshots of resistance mechanisms, with relatively limited attention given to the early, reversible stage of resistance mediated by DTPs (Chavez-Dominguez et al., 2023). Although the role of DTPs as a precursor to acquired resistance is increasingly recognized, the specific molecular features, metabolic dependencies, and precise mechanisms governing the transition from the persister state to fully resistant clones in OSCC remain to be fully elucidated. A more comprehensive understanding of these dynamic trajectories, selection pressures during therapy, and adaptive responses of the TME is still evolving (Ling et al., 2026).

Future research should aim to construct dynamic, cross-scale models of the resistance ecosystem, foster deeper integration of multi-omics data with clinical trajectories, and validate novel combination strategies through prospective clinical trials. Such efforts will be crucial for closing the translational loop from mechanistic discovery to effective clinical intervention.
